# A novel role for ADAMTS13 in hyperfibrinolysis after trauma-induced shock^☆^^☆^

**DOI:** 10.1016/j.bja.2025.07.092

**Published:** 2025-10-06

**Authors:** Pieter H. Sloos, Lisa Vermeersch, Daphne S. Roozendaal, Anne-Sophie Delmote, Cornelis van ‘t Veer, Thomas O. Momanyi, Frida Z. Miranda, Joshua Muia, Claudia Tersteeg, Karen Vanhoorelbeke, Nicole P. Juffermans, Derek J.B. Kleinveld

**Affiliations:** 1 Department of Intensive Care Medicine, location University of Amsterdam, Amsterdam, The Netherlands; 2 Laboratory of Experimental Intensive Care and Anesthesiology, Amsterdam UMC, location University of Amsterdam, Amsterdam, The Netherlands; 3 Laboratory for Thrombosis Research, KU Leuven Campus Kulak Kortrijk, Kortrijk, Belgium; 4 Center for Experimental and Molecular Medicine, location University of Amsterdam, Amsterdam, The Netherlands; 5 Oklahoma State University Center for Health Sciences, Department of Biochemistry and Microbiology, Tulsa, OK, USA; 6 Versiti Blood Research Institute, Milwaukee, WI, USA; 7 Department of Intensive Care Medicine, Erasmus MC, Rotterdam, The Netherlands; 8 Department of Anesthesiology, Erasmus MC, Rotterdam, The Netherlands

**Keywords:** ADAMTS13, coagulopathy, hyperfibrinolysis, trauma, trauma-induced coagulopathy

## Abstract

**Background:**

Trauma-induced coagulopathy with hyperfibrinolysis is associated with high mortality. The protease ADAMTS13 (a disintegrin and metalloprotease with thrombospondin type 1 motifs 13) is cleaved after tissue injury. We analysed the underlying mechanism of ADAMTS13 proteolytic cleavage and the effect of ADAMTS13 on trauma-induced hyperfibrinolysis.

**Methods:**

Thirty-nine trauma patients with shock were stratified based on ADAMTS13 conformation. Mechanisms of ADAMTS13 cleavage were explored *in vitro* using blood from healthy volunteers. The role of ADAMTS13 in hyperfibrinolysis was examined using rotational thromboelastometry (ROTEM) and a fibrin formation assay.

**Results:**

ADAMTS13 circulated in a truncated and hyperactive form in 23% of trauma patients in shock. Truncated ADAMTS13 was associated with a significant increase in hyperfibrinolysis (ROTEM FIBTEM maximum lysis 85 [62–100] *vs* 1 [0–7]%, *P*=0.002) and mortality (44% *vs* 3%, *P*=0.007) compared with patients with a closed ADAMTS13 conformation. *In vitro*, ADAMTS13 was cleaved at its distal self-regulating domains in the presence of tissue plasminogen activator and plasmin, which concentration-dependently increased ADAMTS13 activity. This activation was prevented by tranexamic acid. Hyperactivated ADAMTS13 was associated with hyperfibrinolysis by both ROTEM and fibrin formation assays compared with conditions where ADAMTS13 was inactivated.

**Conclusions:**

During trauma-induced shock, ADAMTS13 can circulate in a truncated and hyperactivated conformation, which is associated with hyperfibrinolysis and mortality. *In vitro*, tissue plasminogen activator and plasmin can truncate ADAMTS13, increasing its activity. Additionally, ADAMTS13 directly contributes to hyperfibrinolysis, and tranexamic acid can prevent ADAMTS13 degradation.

**Institutional ethical review board approval numbers:**

NL58766.018.16, NL74188.018.20.

**Editor’s key points**

• Trauma-induced coagulopathy with hyperfibrinolysis after severe trauma is associated with high morbidity and mortality.
• Mechanisms of ADAMTS13 cleavage and the role of ADAMTS13 in hyperfibrinolysis were examined in blood from 39 trauma patients and healthy controls.
• In trauma-induced shock, the protease ADAMTS13 can be cleaved and hyperactivated by tissue plasminogen activator and plasmin.
• Hyperactivated ADAMTS13 increased fibrinolysis, which was prevented by tranexamic acid, suggesting a possible therapeutic strategy to protecting full-length ADAMTS13 after trauma-induced shock.

Bleeding after trauma is a leading cause of global mortality.^1^, ^2^, ^3^, ^4^ The presence of trauma-induced coagulopathy with hyperfibrinolysis is associated with mortality rates up to 50%.^5^ The main driver of hyperfibrinolysis after trauma is endothelial activation as a result of tissue hypoperfusion,^6^ instigating release of tissue plasminogen activator (tPA) and saturation of plasminogen activator inhibitor 1.^7^^,^^8^ Additionally, endothelial activation results in secretion of von Willebrand factor (vWF) multimers, which facilitate platelet binding to the damaged vascular wall. vWF multimer length is regulated by ADAMTS13 (a disintegrin and metalloprotease with thrombospondin type 1 motifs, member 13), which cleaves vWF multimers into smaller, less haemostatically potent, fragments.

ADAMTS13 circulates in a quiescent, or ‘closed’ conformation in which its activity is self-regulated by its terminal CUB (complement components C1r and C1s, sea urchin protein Uegf, and bone morphogenetic protein-1) domains, which are noncovalently bound to the central spacer domain. Upon binding to VWF, ADAMTS13 changes from a closed to an open conformation, in which the CUB and spacer domains uncouple, activating ADAMTS13.^9^^,^^10^ Recently it has been found that ADAMTS13 can circulate in a full-length but open conformation in patients with immune-mediated thrombotic thrombocytopenic purpura (TTP).^11^ This open ADAMTS13 conformation can be detected *in vitro* by the 1C4 antibody, which binds a cryptic epitope in the spacer domain of ADAMTS13. However, truncation of ADAMTS13 at its distal thrombospondin or CUB domain also leads to exposure of the 1C4 epitope, and is associated with increased ADAMTS13 activity.^9^ Truncation of ADAMTS13 has been observed in some patients with congenital TTP, but might also play an important role in other disease states.^12^

Early after trauma, circulating concentrations of vWF increase and remain elevated up to 3 days after injury.^13^ Conversely, ADAMTS13 antigen and activity are generally decreased after trauma, associated with an increased risk of microvascular thrombosis and organ dysfunction.^13^, ^14^, ^15^, ^16^ In severely injured patients, ADAMTS13 activity can also be increased.^17^ It is currently unknown which mechanisms are responsible for changes in ADAMTS13 activity after trauma. In some patients, ADAMTS13 might get truncated resulting in decreased full-length ADAMTS13 antigen but increased activity.^9^^,^^17^ Both thrombin and plasmin have the capacity to cleave ADAMTS13 *in vitro*, but their effect on ADAMTS13 activity after trauma is unknown.^18^^,^^19^ Likewise, the effects of increased ADAMTS13 activity during hyperfibrinolysis are unclear.

This study aimed to (1) analyse the conformation and activity of ADAMTS13 in trauma-induced shock; (2) determine how plasmin and thrombin influence full-length ADAMTS13; and (3) determine whether hyperactivated ADAMTS13 can accelerate fibrinolysis. We hypothesised that ADAMTS13 is cleaved into a truncated hyperactivated form during severe traumatic shock, and that hyperactivated ADAMTS13 accelerates fibrinolysis.

## Methods

### Trauma cohort study

Activation of Coagulation and Inflammation in Trauma-III (ACIT-III) was an international prospective cohort study in which trauma patients were included between 2012 and 2018 (ethical approval number NL58766.018.16). To test our hypothesis, patients from the Amsterdam UMC (The Netherlands) who met the ACIT-III inclusion criteria (Supplementary Table 1) were included if they had signs of shock (base deficit [BD] ≥5 mM or activation of the massive transfusion protocol [MTP, including balanced ratio of blood products and early administration of tranexamic acid, TXA, 1 g]). Additionally, a random selection of nine patients with a BD <5 mM, an injury severity score of <16, and without activation of the MTP were included. Deferred consent was obtained from all patients or their relatives within 24 h after inclusion. Blood was collected within 2 h of traumatic injury in trisodium citrate. Rotational thromboelastometry (ROTEM; Tem International GmbH, Munich, Germany) Delta EXTEM and FIBTEM were performed to quantify fibrinolysis. The remaining citrated blood was centrifuged twice at 1750×*g* for 10 min at 18°C, and plasma was stored at −80°C for later analysis of ADAMTS13 antigen, conformation, and activity (Supplementary Table 2).

#### ADAMTS13 antigen, conformation index, and activity

Full-length ADAMTS13 antigen was measured using enzyme-linked immunosorbent assay (ELISA) as described.^11^^,^^20^ ADAMTS13 conformation (i.e. exposure of the cryptic epitope on the spacer domain) was determined with ELISA using the capturing antibody 1C4 that binds to a cryptic epitope in the spacer domain of ADAMTS13. This is possible when ADAMTS13 is in an open or truncated structure. To calculate the conformational index (CI), ADAMTS13 detected with the 1C4 antibody was corrected for full-length ADAMTS13 antigen levels and then normalised using pooled reference plasma that was pre-incubated with the 17G2 antibody, which binds the CUB1 domain and forces an open ADAMTS13 conformation. Patients with a CI <0.5 and a CI >0.5 after addition of 17G2 were classified as having a closed and full-length ADAMTS13.^11^ Patients who had either a CI >0.5 or a CI <0.5 after addition of 17G2 were classified as having truncated ADAMTS13. ADAMTS13 activity was measured using a fluorescence resonance energy transfer (FRET) assay, with the FRETS-rVWF71 fragment.^21^ More details can be found in the Supplementary Methods.

#### *In vitro* study

For the *in vitro* study, venous blood was withdrawn from healthy male volunteers after institutional ethical approval (NL74188.018.20) and informed consent from volunteers. Blood was collected in trisodium citrate or in glass serum tubes (without additives). To obtain plasma or serum, blood was centrifuged twice at 2500×*g* for 15 min at 18°C and snap-frozen in liquid nitrogen for storage at −80°C for later analysis. More details can be found in the Supplementary Methods.

#### Incubation experiments

Part of the whole blood and plasma was incubated immediately after blood withdrawal for 30 or 120 min at 37°C. Plasma was incubated with plasmin (Sigma Aldrich, St Louis, MO, USA; 30–1200 mU ml^−1^), thrombin (Sigma Aldrich; 0.5 U ml^−1^ and 1.5 U ml^−1^), urokinase-type plasminogen activator (uPA; Devrimed, Sint Annaparochie, the Netherlands; 100 IU ml^−1^ and 300 IU ml^−1^) or tPA (Actilyse, Boehringer Ingelheim, Ingelheim am Rhein, Germany; 150 IU ml^−1^ and 1200 IU ml^−1^). Whole blood without anticoagulant (glass serum tube) was incubated with tPA (150 IU ml^−1^ and 1200 IU ml^−1^) or tissue factor (TF; Innovin Dade, Siemens Healthcare, Erlangen, Germany; 1.1 pM and 110 pM). In some tPA conditions, TXA (Mylan, Morgantown, WV, USA; 0.2 mg ml^−1^) or aprotinin (Abcam, Cambridge, UK; 250 KIU ml^−1^) were added during incubation. After incubation, D-phenylalanyl-L-prolyl-L-arginine chloromethyl ketone 100 μM (PPACK; Cayman Chemical, Ann Arbor, MI, USA) and aprotinin 250 KIU ml^−1^ were added to inhibit any remaining thrombin and plasmin, respectively. Whole blood and plasma were then processed as described and stored at −80°C.

Full-length ADAMTS13 antigen and ADAMTS13 activity were determined as described. An additional ELISA was performed using the biotinylated 15D1 detection antibody targeting the spacer domain, thus measuring both full-length and truncated ADAMTS13. Further details are described in the Supplementary Methods and Supplementary Fig. 1.

#### Rotational thromboelastometry

For the *in vitro* study, whole blood from six healthy volunteers was incubated with 40 μg ml^−1^ of the ADAMTS13 opening antibody 17G2 or 40 μg ml^−1^ of the ADAMTS13 inactivating antibody 3H9 in the presence of tPA 150 IU ml^−1^ 20 min before assay. ROTEM Delta EXTEM was then performed according to the manufacturer’s guidelines.

#### Fibrin formation assay

Plasma from 10 healthy volunteers was added to a low-binding, flat-bottomed 96-well plate (Corning, NY, USA). HEPES-buffered saline (HBS; HEPES 25 mM, NaCl 137 mM, pH 7.4), supplemented with tPA 150 IU ml^−1^, recombinant ADAMTS13 2 μg ml^−1^, 3H9 40 μg ml^−1^ or 17G2 40 μg ml^−1^ (all final concentrations), was added and incubated for 5 min at room temperature. Fibrin formation was initiated with addition of HBS coagulation buffer consisting of CaCl_2_ 15 mM, phospholipids 4 μM (DOPE:DOPS:DOPC, 50:30:20; Polarlipids, Alabaster, AL, USA) and tissue factor 1.1 pM in HBS. Final volume per well was 70 μl, consisting of >80% plasma. Absorbance (i.e. turbidity) was measured at 405 nm every 15 s for 40 min using the SpectaMaxM2 plate reader (Molecular Devices, San Jose, CA, USA). Outcomes included 50% clot lysis time, optical density at 20 min, and the area under the curve (Supplementary Fig. 2).

### Statistical analysis

All data were analysed using SPSS version 25.0 (IBM, Armonk, NY, USA). Graphs were made using GraphPad Prism version 9.0 (GraphPad Software, San Diego, CA, USA). Data were analysed as nonparametric and presented as median with interquartile range (IQR) unless stated otherwise. Independent data were analysed with the Kruskal–Wallis test with *post hoc* Dunn’s test. For the *in vitro* study, data were analysed with a Friedman two-way analysis of variance by ranks with pairwise comparisons adjusted by the Bonferroni correction or by the Wilcoxon signed rank test. Binominal data were analysed with Fisher’s exact test or the χ^2^ test. A *P*-value <0.05 was considered to be statistically significant.

## Results

### Truncated ADAMTS13 is associated with hyperfibrinolysis in trauma patients with shock

**Fig 1:**
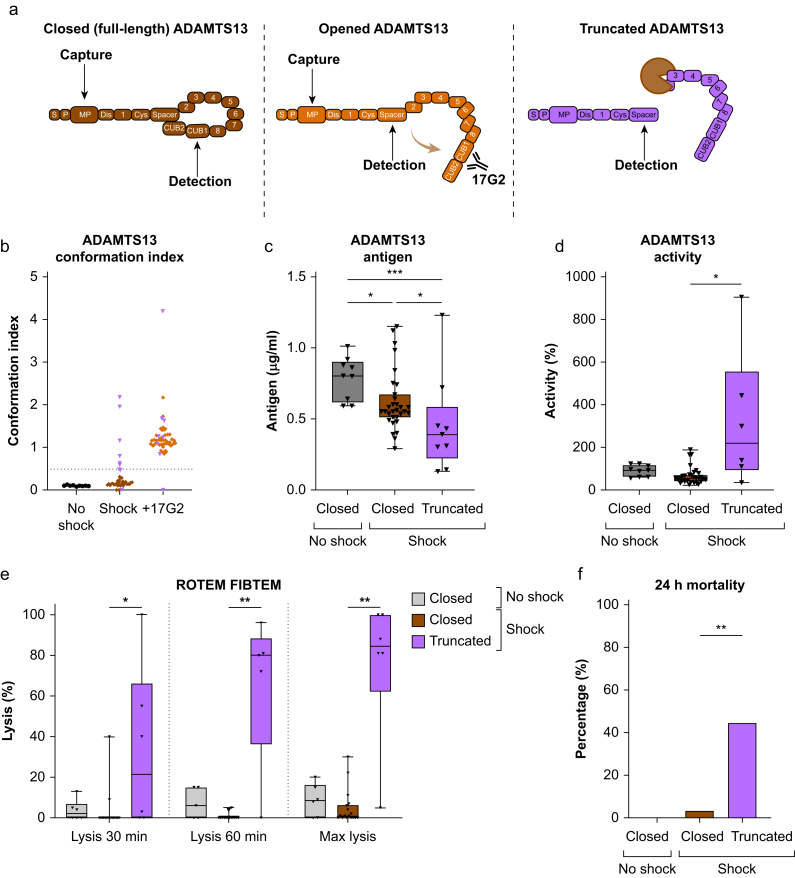
Truncated ADAMTS13 is associated with hyperfibrinolysis and mortality in patients with trauma-induced shock. (a) Normally ADAMTS13 circulates in a closed conformation, where its spacer domain is covered by the CUB domains. Detection of the spacer domain is only possible when ADAMTS13 undergoes an antibody-induced conformational change, or when ADAMTS13 is truncated distal to the spacer domain. (b) ADAMTS13 conformational index in trauma patients without shock and with shock (base deficit ≥5 mM or activation of massive transfusion protocol). Truncated ADAMTS13 is defined as a conformational index of >0.5, or a conformational index <0.5 after incubation with the opening antibody 17G2. (c) Full-length ADAMTS13 antigen in trauma patients without shock and with shock, further stratified by ADAMTS13 conformational state (closed *vs* truncated). (d) Truncated ADAMTS13 is associated with a significant increase ADAMTS13 activity compared with a closed ADAMTS13 conformation. (e) Truncated ADAMTS13 is associated with a significant increase in fibrinolysis measured by the rotational thromboelastometry (ROTEM) FIBTEM assay. (f) Truncated ADAMTS13 is associated with significantly increased mortality compared with a closed ADAMTS13 conformation. Data are presented as boxplot with all data points. ∗*P*< 0.05, ∗∗*P*<0.01, ∗∗∗*P*<0.001.

We enrolled 39 trauma patients with shock and nine patients without shock (Table 1). Measurement of the ADAMTS13 CI revealed that in nine patients (23% of patients in shock) truncated ADAMTS13 circulated (Fig. 1a and b). Eight of these patients had a CI of >0.5. One patient had a CI of 0 both with and without incubation with the opening antibody 17G2, suggesting truncation proximal to the spacer domain. We confirmed that exposure of the cryptic domain was not as a result of autoantibodies against ADAMTS13 (Supplementary Table 3). All other patients had closed and full-length ADAMTS13. Full-length ADAMTS13 antigen was lower in patients with a truncated ADAMTS13, compared with patients with closed ADAMTS13 (0.49 [0.22–0.59] *vs* 0.55 [0.51–0.67] μg ml^−1^, *P*=0.046; Fig. 1c). However, truncated ADAMTS13 was associated with higher ADAMTS13 activity compared with closed ADAMTS13 (219 [91–559] *vs* 54 [39–71]%, *P*=0.011; Fig. 1d).

**Table 1:** Characteristics of trauma patients upon emergency room arrival. Trauma patients were stratified based on the presence of shock (BD >5 or activation of the massive transfusion protocol [MTP]). Patients in shock were further stratified based on ADAMTS13 conformation (closed or truncated). BD, base deficit; Hb, haemoglobin; ISS, injury severity score; TBI, traumatic brain injury defined as an abbreviated injury scale (AIS) in head/neck of ≥3; TXA, tranexamic acid; vWF, von Willebrand factor. ∗*P*-value based on a comparison between the closed ADAMTS13 group and the truncated ADAMTS13 group (Mann–Whitney *U*-test or χ^2^ test).

Patient characteristics	No shock	Shock		
	Closed ADAMTS13	Closed ADAMTS13	Truncated ADAMTS13	*P*-value∗
	(*N*=9)	(*N*=30)	(*N*=9)	
Patient characteristics				
Age (yr)	52 (48–60)	51 (35–73)	37 (22–79)	0.35
Male sex, *n* (%)	8 (89)	24 (80)	6 (67)	0.41
Blunt injury, *n* (%)	8 (89)	29 (97)	7 (78)	0.13
ISS	5 (3–9)	22 (15–35)	24 (17–28)	0.92
TBI, *n* (%)	1 (11)	11 (39)	4 (57)	0.71
TXA received before blood withdrawal, *n* (%)	0 (0)	16 (53)	5 (56)	0.91
Crystalloids (ml)	1000 (1000–2300)	2000 (900–3400)	1000 (0–2700)	0.02
Packed red blood cells (units)	0 (0–0)	0 (0–4)	0 (0–3)	0.62
Plasma (units)	0 (0–0)	0 (0–1)	0 (0–2)	0.50
Platelets (units)	0 (0–0)	0 (0–0)	0 (0–0)	0.57
Shock				
Lactate (mM)	2.1 (1.4–2.5)	5.4 (3.1–8.5)	4.6 (3.1–7.1)	0.72
Base deficit (mM)	-1.9 (-2.5 to -0.2)	6.6 (5.2–10.7)	7.7 (7–23)	0.54
MTP activated, *n* (%)	0 (0)	8 (27)	3 (33)	0.69
Haematology				
Hb (g dl^−1^)	14.2 (13.7–15.2)	12.2 (10.5–13.7)	13.5 (11.6 –14.4)	0.23
Platelets (×10^9^ L^−1^)	210 (195–237)	239 (184–267)	210 (195–236)	1.00
vWF antigen	1270 (1040 –1760)	1760 (1170 –2570)	2050 (730–3880)	0.92
Outcomes				
Mortality, 24 h, *n* (%)	0 (0)	1 (3)	4 (44)	0.01
Mortality, 28 days, *n* (%)	0 (0)	6 (20)	5 (56)	0.29

Shock patients with truncated ADAMTS13 had an increase in fibrinolysis compared with those with full-length ADAMTS13 (ROTEM FIBTEM maximum lysis: 85 [62–100] *vs* 1 [0–7]%, *P*=0.002; Fig. 1e). Clotting time, maximum clot firmness, and maximum lysis in the EXTEM assay were also impaired in patients with truncated ADAMTS13 compared with patients with closed ADAMTS13 (Supplementary Table 4). The percentage of patients who received TXA did not differ between groups. In patients who received TXA, ADAMTS13 activity did not differ from those who did not (Table 1 and Supplementary Fig. 3). Patients with truncated ADAMTS13 had increased early mortality compared with patients with full-length ADAMTS13 (44% *vs* 3%, *P*=0.007; Fig. 1f and Supplementary Table 4).

### ADAMTS13 is truncated and activated by tissue plasminogen activator and plasmin

**Fig 2:**
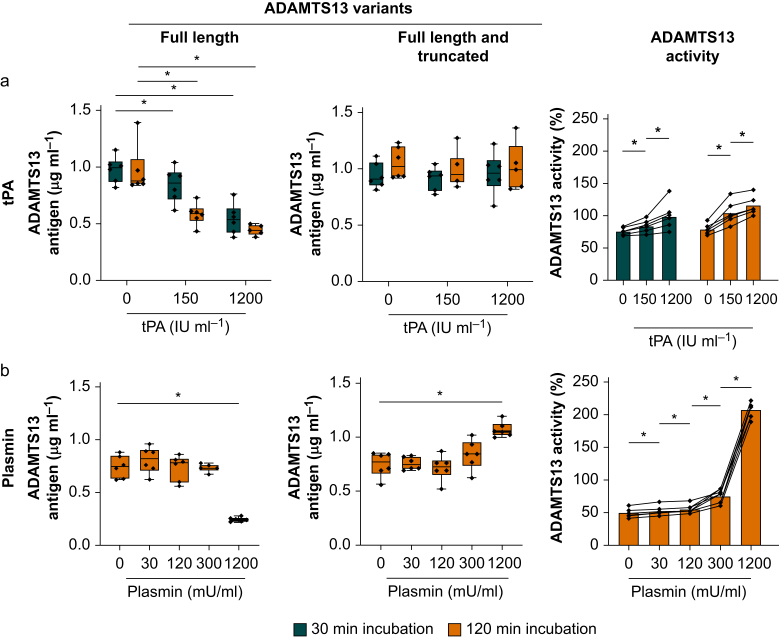
ADAMTS13 is truncated in the presence of tissue plasminogen activator and plasmin, increasing its activity. (a) Incubation of whole blood with tissue plasminogen activator (tPA) reduced full-length ADAMTS13 antigen in a concentration-dependent manner, producing truncated ADAMTS13 with increased activity. (b) Incubation of plasma with plasmin resulted in truncated ADAMTS13 only with the highest dose of plasmin. Incubation with all plasmin concentrations was associated with increased ADAMTS13 activity. ADAMTS13 antigen is presented as boxplots, and ADAMTS13 activity is presented as before–after plots with samples from the same individual volunteer connected. All data points are shown. ∗*P*<0.05.

**Fig 3:**
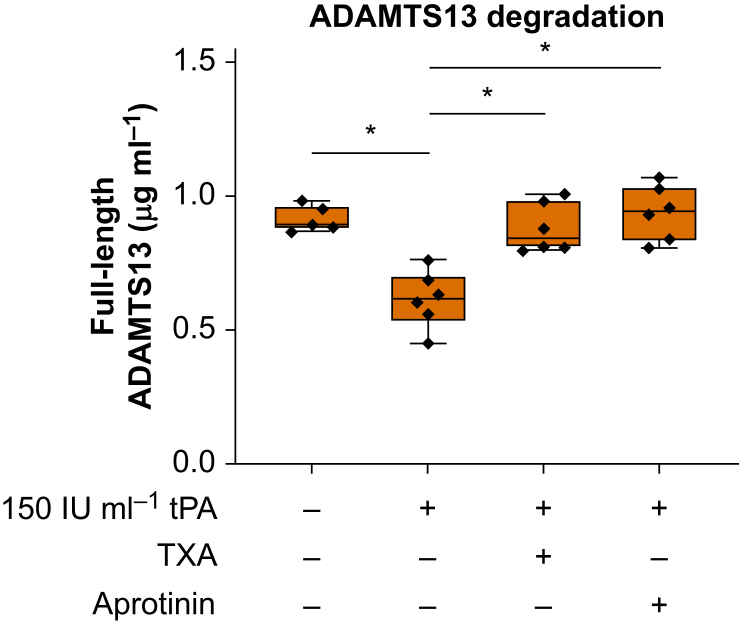
Degradation of ADAMTS13 is prevented by tranexamic acid and aprotinin *in vitro*. tPA, tissue plasminogen activator; TXA, tranexamic acid. Data are presented as boxplot with all data points. ∗*P*<0.05.

Incubation of whole blood with tPA resulted in a significant decrease in ADAMTS13 antigen detected with the 17G2 antibody, but not with the 15D1 antibody, indicating cleavage of ADAMTS13 distal to the spacer domain (i.e. truncated ADAMTS13; Fig. 2a). Incubation of plasma with plasmin also generated truncated ADAMTS13, but only significant at a plasmin concentration of 1200 IU ml^−1^ (Fig. 2b). ADAMTS13 activity increased in a concentration-dependent manner with both tPA and plasmin (Fig. 2a and b). TXA and aprotinin prevented cleavage of ADAMTS13 in the presence of tPA (Fig. 3). Incubation with uPA in citrated plasma had similar effects on ADAMTS13 antigen as tPA (Supplementary Fig. 4). However, incubation with tPA in citrated plasma, in which plasmin is not formed because of lack of fibrin formation, did not result in truncation of ADAMTS13 (Supplementary Fig. 5).

**Fig 4:**
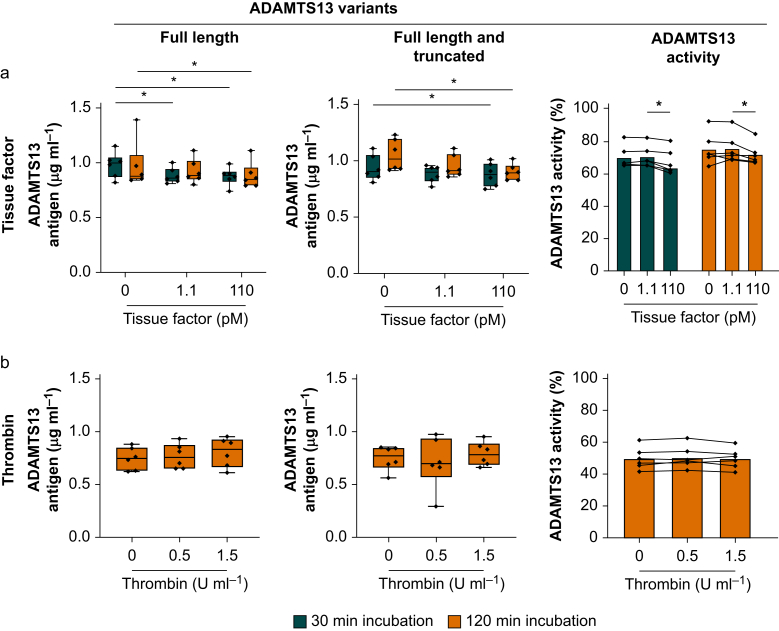
Effects of tissue factor and thrombin on ADAMTS13 degradation. (a) Incubation of whole blood with tissue factor (TF) slightly, but significantly, decreased ADAMTS13 antigen. ADAMTS13 activity was decreased only with the highest concentration of TF. (b) Incubation of plasma with thrombin did not affect ADAMTS13 antigen or activity. ADAMTS13 antigen is presented as boxplot and ADAMTS13 activity is presented as a before–after plot with samples from the same individual volunteer connected. All data points are shown. ∗*P*<0.05.

Incubation of whole blood with tissue factor was associated with a slight, but significant, decrease in ADAMTS13 detected with both the 17G2 antibody and the 15D1 antibody compared with control, suggesting degradation of both full-length and truncated ADAMTS13. Concordantly, a slight but significant decrease in ADAMTS13 activity was seen (Fig. 4a). Direct incubation with thrombin in plasma did not result in significant ADAMTS13 degradation or changes in ADAMTS13 activity (Fig. 4b).

### Hyperactivated ADAMTS13 enhances fibrinolysis *in vitro*

**Fig 5:**
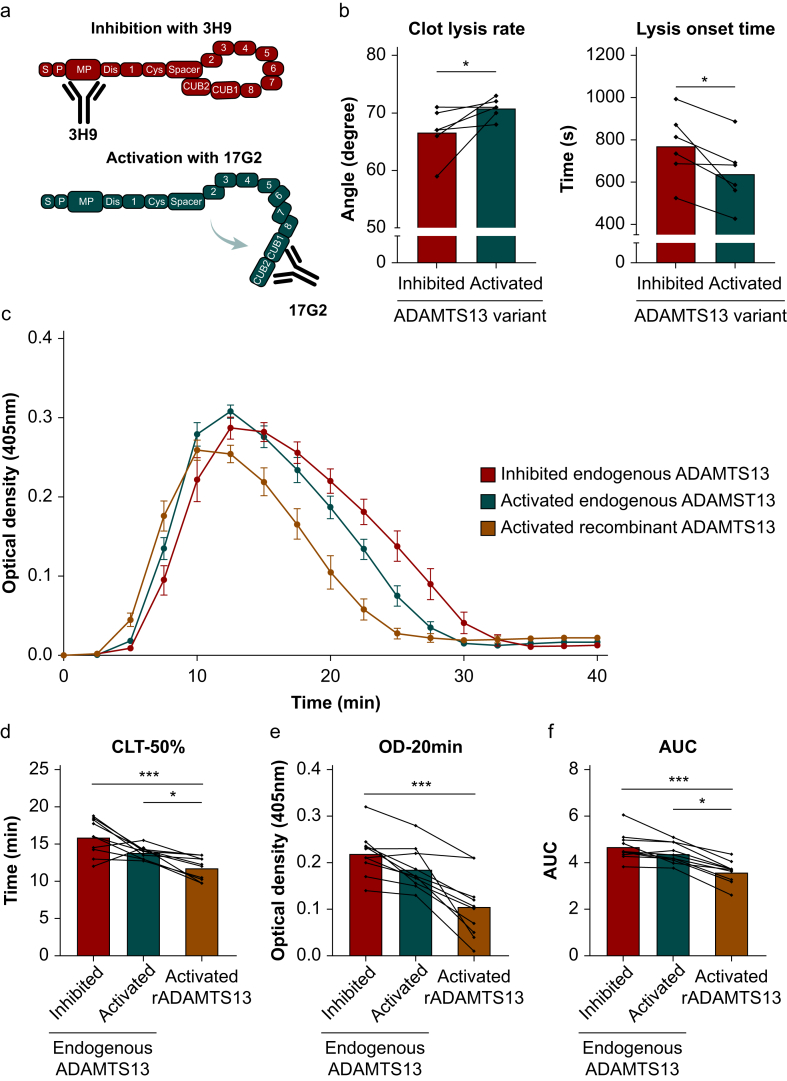
Hyperactive ADAMTS13 accelerates hyperfibrinolysis *in vitro*. (a) ADAMTS13 can be inhibited or activated by the antibodies 3H9 and 17G2, respectively. (b) ROTEM EXTEM. Activated ADAMTS13 is associated with decreased lysis onset time and increased clot lysis in the presence of tissue plasminogen activator (tPA) 150 IU ml^−1^ compared with inhibited ADAMTS13. (c, d) Fibrin formation assay. Recombinant ADAMTS13 (rADAMTS13) activated with 17G2 is associated with a decreased clot lysis time 50% (CLT-50%), decreased optical density at 20 min (OD-20 min), and decreased area under the curve (AUC) in the presence of tPA 150 IU ml^−1^, compared with inhibited ADAMTS13. Data presented as mean with all data points or mean with standard error. ∗*P*<0.05, *∗∗∗P*<0.001.

In ROTEM EXTEM, addition of the ADAMTS13 opening antibody 17G2 (Fig. 5a) resulted in a 20 (9–33)% relative decrease in lysis onset time (*P*=0.028) and a 4 (1–11)% relative increase in clot lysis rate compared with the inhibiting antibody 3H9 (*P*=0.043; Fig. 5b). The same experiment was conducted in a plasma fibrin formation assay (Fig. 5c and d). Addition of 17G2 to plasma did not increase tPA-induced fibrinolysis compared with addition of 3H9. However, combining 17G2 with recombinant ADAMTS13 (rADAMTS13) 2 μg ml^−1^ resulted in a 28 (18–32)% reduction in clot lysis time (*P*<0.001), a 54 (43–77)% reduction in the optical density at 20 min (*P*<0.001), and a 24 (16–30)% reduction in the area under the curve compared with addition of 3H9 (*P*<0.001).

Using isolated proteins, activated rADAMTS13 was not able to cleave fibrinogen directly (Supplementary Fig. 6). However, co-incubation of rADAMTS13 with plasmin (12 mU ml^−1^ to 1200 mU ml^−1^) increased fibrinogen degradation products in conditions with activated rADAMTS13 compared with inhibited rADAMTS13.

## Discussion

We identified a novel role for ADAMTS13 in severe traumatic injury. In 23% of trauma patients in shock, ADAMTS13 was truncated, which was associated with increased ADAMTS13 activity, hyperfibrinolysis, and mortality. Both tPA and plasmin can truncate ADAMTS13 in its autoregulatory domain, increasing its activity. Moreover, hyperactivated ADAMTS13 accelerated tPA-induced lysis in plasma and in whole blood assays.

Temporal changes in coagulation are characteristic of trauma-induced coagulopathy. We focused on the bleeding or acute phase of injury, characterised by hypocoagulability and hyperfibrinolysis.^5^ We found a discrepancy between low ADAMTS13 antigen and high ADAMTS13 activity in 23% of severely injured trauma patients with shock. In those patients, ADAMTS13 circulated in a truncated and hyperactivated form. Truncated ADAMTS13 was further associated with increased fibrinolysis and mortality, revealing a potential novel therapeutic target.

These results contrast with a previous report in trauma patients showing that ADAMTS13 antigen and activity are decreased after trauma and are associated with blood loss and poor outcomes.^13^ However, our study focused specifically on trauma patients with shock, which could account for the differences. Differences in the timing of blood sampling might also explain part of the discrepancy. As time progresses, ADAMTS13 antigen and activity might decrease, which can be associated with thrombotic complications and organ dysfunction.^16^^,^^17^

Based on the findings in this trauma cohort, we postulate that either increased tPA release or excessive tissue factor and thrombin release are responsible for cleavage of ADAMTS13. In whole blood and plasma, we observed concentration-dependent cleavage of ADAMTS13 between the spacer and CUB1 domain in the presence of tPA, uPA, and plasmin, producing truncated ADAMTS13 variants with concomitant increases in activity. Incubation of citrated plasma with tPA does not result in plasmin formation as tPA-mediated plasminogen conversion requires fibrin formation. Under these conditions tPA did not affect ADAMTS13, which further points to a direct effect of plasmin on ADAMTS13. This is in line with a previous study in which addition of streptokinase to human plasma resulted in a decrease in full-length ADAMTS13 and the appearance of smaller ADAMTS13 fragments and ∼20% increased ADAMTS13 activity.^18^ Other studies have found with recombinant proteins, plasmin cleaved ADAMTS13 into smaller fragments with reduced activity in proteolysing vWF.^19^^,^^22^^,^^23^ The decrease in full-length ADAMTS13 we observed in the presence of tPA was gradual, increasing with incubation time and tPA concentration. With plasmin, we observed a more precipitous decrease in full-length ADAMTS13 and increase in ADAMTS13 activity, but only at the highest plasmin concentrations. There is likely a continuous conversion of plasminogen resulting in gradual saturation of anti-plasmin in the presence of tPA, whereas with plasmin addition, anti-plasmin remains sufficient to counteract the proteolytic effect of lower plasmin concentrations.

Our *in vitro* data also show that TXA and aprotinin can reverse the effect of tPA on ADAMTS13 cleavage. In the clinical study, there was no significant difference between the percentage of patients who received TXA. More than half of patients with truncated ADAMTS13 were pretreated with TXA 1 g before blood sampling, which implies that in a subset of patients the dose and perhaps timing of treatment was not adequate to protect ADAMTS13 from truncation, or that there are additional (plasmin-independent) mechanisms explaining ADAMTS13 degradation. Our data raise the question of whether (a subset of) patients in shock should receive a higher dose of TXA, which warrants further investigation.^4^^,^^24^

Traumatic injury is characterised by increased tissue factor expression and thrombin generation.^25^ Our results show that in the presence of tissue factor, ADAMTS13 is minimally cleaved other than in the autoregulatory domains, with corresponding reductions in activity. However, the decrease in activity was relatively small and might not have clinical implications. Incubation with thrombin did not cleave ADAMTS13 degradation in our experiments. Previous studies have shown that thrombin cleaves ADAMTS13 at the CUB1-2 domain when using recombinant proteins in isolated conditions with mixed results on ADAMTS13 activity.^19^^,^^22^ In a follow-up study, thrombin did not cleave ADAMTS13 when fibrinogen-depleted plasma was incubated with thrombin, perhaps because of low affinity of thrombin for ADAMTS13.^26^

Truncation of ADAMTS13 during hyperfibrinolysis could have several clinical consequences. Although the distal thrombospondin and CUB domains of ADAMTS13 are not required for cleavage within the vWF A2 domain, these domains increase cleavage efficiency of full-length vWF and regulation of proximal catalytic domains.^9^^,^^27^, ^28^, ^29^, ^30^ We found increased tPA-induced fibrinolysis in both whole blood and plasma with hyperactivated ADAMTS13 compared with conditions in which ADAMTS13 was inhibited. Additionally, we showed that hyperactivated ADAMTS13 leads to an increase in fibrinogen degradation products in isolated conditions, but only in the presence of plasmin. These data suggest that cleavage of ADAMTS13 by plasmin is a prerequisite for degradation of fibrinogen by ADAMTS13. Earlier reports suggest that truncated ADAMTS13 variants can degrade fibrinogen directly or indirectly through plasminogen activation.^27^^,^^31^ Overall, our data support a causal relationship between hyperactivated ADAMTS13 and hyperfibrinolysis, in line with our observation in the trauma cohort.

This study has several limitations. Firstly, the findings of the cohort study are inherently associative, in which the causal relationship between a truncated ADAMTS13 and the progression of hyperfibrinolytic coagulation profiles cannot be definitively established. However, we have further explored this relationship in *in vitro* models, identifying a potential mechanism that strengthens these observations. A lack of plasma sample volume in some of the most severely injured trauma patients and in the volunteers constrained our ability to measure additional coagulation and fibrinolytic makers, which could have enhanced the robustness of our findings. One of the limitations of ROTEM is the lack of sensitivity and specificity to detect fibrinolysis. Reduced maximum clot amplitude in ROTEM could be attributable to clot lysis or increased clot retraction.^32^ Despite these limitations, it is noteworthy that ROTEM, used as the primary point-of-care assay for trauma-induced fibrinolysis, is widely recognised and utilised in clinical settings, indicating its clinical relevance.

In conclusion, ADAMTS13 can circulate in a truncated, hyperactivated form in a subset of trauma patients with shock, which is associated with hyperfibrinolysis and mortality. tPA-induced plasmin formation truncates ADAMTS13, increasing its activity. Truncated ADAMTS13 might lose its vWF specificity and accelerate fibrinogen degradation after trauma, which might be prevented with tranexamic acid. These results broaden our understanding of ADAMTS13 truncation after trauma and add a novel mechanism of trauma-induced hyperfibrinolysis. Future studies should explore whether treatment strategies aimed at protecting full-length ADAMTS13 after trauma-induced shock improve patient outcome.

## Authors’ contributions

Study design: PHS, KV, NPJ, DJBK

Performed *in vitro* experiments, data analysis: PHS

Writing original draft: PHS, DJBK

Final editing: PHS, DJBK

Contributed to the ADAMTS13 measurements: LV, DSR, A-SD

Coagulation assay setup: CvV

Data interpretation: CvV, TOM, FZM, JM, CT, KV, NPJ, DJBK

Provided reagents for ADAMTS13 assays: TOM, FZM, JM

ADAMTS13 data analysis: TOM, FZM, JM, CT, KV

Study supervision: NPJ, DJBK

Revised the draft of the manuscript and approved the final version: all authors

## Data availability

Original data are available upon reasonable request from the corresponding author. Individual participant data cannot be shared.

## Funding

Funding from the Prof. Heimburger Award 2021 (to PHS), the ‘Fonds voor Wetenschappelijk Onderzoek, Vlaanderen’ (G090120N and G009923N to KV), the US National Institutes of Health, National Institute of General Medical Sciences (grant R35 GM142936 to JM), and the Amsterdam University Fund and the European Society for Anaesthesiology and Intensive Care (ESAIC) Research Project Grant 2021 (ESAIC_GR_2021_DK to DJBK).

## Declaration of interests

JM holds the patent ‘Fluorogenic substrate for ADAMTS13’ (US 8663912), issued to Washington University. All other authors declare that they have no conflicts of interest.
