# Supplementary material for: A novel role for ADAMTS13 in hyperfibrinolysis after trauma-induced shock☆
Source: Br J Anaesth. 2025 Oct 6;136(1):107–16. doi: 10.1016/j.bja.2025.07.092 (PMC13269631; doi:10.1016/j.bja.2025.07.092)
Supplement: Multimedia component 1 [file mmc1.docx]

**Supplemental methods & results**

**ADAMTS13 measurements**

*ADAMTS13 conformation to determine full-length and truncated ADAMTS13*

The conformation of ADAMTS13 (i.e. exposure of the cryptic epitope on the spacer domain) was determined by enzyme-linked immunosorbent assay (ELISA) as previously described.(1) In short, a 96-well plate was coated with the ADAMTS13 antibody 1C4, which recognizes a cryptic epitope in the spacer domain. The captured ADAMTS13 was detected with a biotinylated 3H9 antibody, which binds to the proximal metalloprotease (MP) domain of ADAMTS13. After addition of horseradish peroxidase (HRP)-labelled streptavidin, o-phenylenediamine dihydrochloride (OPD) and H_2_O_2_, the optical density (OD) was determined at 490nm (OD_490_).

The ELISA was also performed after incubating the samples with the CUB1 antibody 17G2, which induces an open ADAMTS13 conformation. As a reference, healthy human control plasma was also incubated with the 17G2 antibody.

The optical density data from the ELISA is expressed as a conformation index (CI). To calculate this, the OD_490_ values of the conformation ELISA from the trauma patients were first corrected for ADAMTS13 antigen levels, as higher antigen levels result in higher OD_490_ values. OD_490_ values were then normalized to the OD_490_ values of the references plasma that was pre-incubated with 17G2.

In this study, a CI of > 0.5 is defined as a truncated ADAMTS13 while a CI of < 0.5 is defined as a full length ADAMTS13. Additionally, if ADAMTS13 could not be captured by mAb 1C4 (CI < 0.5) and could not be opened by 17G2 (i.e. CI after addition of 17G2 was < 0.5) patients were also classified as having a truncated ADAMTS13.

The threshold of 0.5 for the CI was chosen based on incubating healthy human plasma with 17G2 which guarantees exposure of the cryptic domain.(1) The conformation index of these samples ranged from 0.5 until 1.8. Without 17G2, the conformation index ranges between 0.05 and 0.23. As 0.5 was the lowest value obtained with 17G2, we choose this as the cutoff value.

*ADAMTS13 antigen*

ADAMTS13 antigen was determined as previously described.(1) A 96-well plate was coated overnight at 4°C with 5 µg/ml of the anti-human ADAMTS13 (MP domain) antibody 3H9 diluted in carbonate/bicarbonate buffer. The plate was subsequently blocked for 2 hours at room temperature with a solution of 3% dried milk powder in phosphate buffered saline (PBS). Next, a serial dilution of the human plasma samples (starting dilution of 1/100) was added and samples were incubated for 1.5 hours at 37°C. Captured ADAMTS13 was detected using either the biotinylated anti-ADAMTS13 CUB1 antibody 17G2 or the biotinylated anti-spacer domain antibody 15D1 to detect full-length and truncated ADAMTS13 variants respectively (1.5 µg/ml each, incubation for 1 hour at room temperature). This was followed by a 1 hour incubation at room temperature with HRP-streptavidin (1/10,000; Roche Diagnostics).

A coloring solution of OPD and H_2_O_2_ was added and the reaction was stopped using 1 M H_2_SO_4_. The OD of the samples was measured using a spectrophotometer at 490 nm. A dilution series of a normal human plasma pool (NHP, plasma from ≥ 20 healthy donors, set at 100%) was used as a reference curve, from which the ADAMTS13 antigen levels were calculated.

*ADAMTS13 activity*

ADAMTS13 activity was assessed by diluting plasma samples (1:12) in 1 μM fluorogenic FRETS-rVWF71 substrate buffer (50 mM HEPES, 1 μM ZnCl_2_, 5 mM CaCl_2_, 150 mM NaCl, 1 mg/ml bovine serum albumin]) in a 96-well plate (Greiner Bio-One). Final volume was 200 μL. The plate was incubated at 30°C and fluorescence intensity was detected for 30 cycles of 2 minutes using a FLUOstar Optima reader (BMG Labtech GmbH) at 620 nm excitation and 660 nm emission wavelengths. The detected fluorescence signal, which is directly proportional to the cleavage of FRETS-rVWF71 substrate by ADAMTS13, was plotted as a function of time. The slope of the linear part was used to calculate the proteolysis rate. A reference curve was obtained from the slopes of different NHP dilutions (1:7 to 1:39 dilutions), allowing the estimation of relative ADAMTS13 activity (with ADAMTS13 activity in NHP defined as 100%). A 1:7 dilution of NHP in presence 10 μg/ml of the inhibitory anti-ADAMTS13 antibody 3H9 was used as a negative control.

*Fibrinogen degradation*

Purified fibrinogen (Fibryga, Octapharma; 2 mg/ml) was incubated with increasing concentrations of plasmin (0 – 1200 mU/ml) and 5 μg/ml rADAMTS13 (R&D systems) with either 40 μg/ml 17G2 or 3H9 antibody for 30 minutes at room temperature, to activate or inhibit ADAMTS13, respectively. Reactions were stopped with the addition of sample buffer (62.5 mM Tris-HCl [pH 6.8], 25% glycerol, 2% SDS, 0.01% bromophenol blue) and samples were heated for 10 minutes at 70°C. Samples were then separated by SDS-PAGE on a 6% acrylamide (Tris-HCl) gel. The gel was washed three times with purified water (Merck Millipore) and stained for one hour with Coomassie Brilliant Blue (BioRad) at room temperature. Excess staining was removed by soaking in purified water for one hour, after which the gel was scanned using the Odyssey DLx Near-Infrared Fluorescence Imaging System at 700 nm and analyzed using the LI-COR image software.

**References**

1. Roose E, Schelpe AS, Joly BS, Peetermans M, Verhamme P, Voorberg J, et al. An open conformation of ADAMTS-13 is a hallmark of acute acquired thrombotic thrombocytopenic purpura. Journal of Thrombosis and Haemostasis. 2018;16(2):378-88.

**Supplemental Table 1. In-/exclusion criteria ACIT-III**

| Inclusion criteria |
| --- |
| Adult trauma patients were enrolled if they sustained a blunt or penetrating trauma and for whom the trauma team was activated, with at least one of the following clinical parameters:   - Respiratory rate < 10 or > 25 times per minute - Heart rate ≥120 beats per minute - Systolic blood pressure < 90 mmHg - Oxygen saturation < 90% - Estimated blood loss ≥ 500 ml - Glasgow Coma Score ≤ 13 or abnormal pupil size and/or reaction   Or clinical signs of at least one of the following diagnoses:   - Femur fracture - Signs of flail thorax/pneumothorax/hematothorax or multiple rib fractures - Signs of significant abdominal injury - Pelvic fracture - Spine injury |
| Exclusion criteria |
| - Age < 18 years old - Patients transferred from other hospitals - Patients presenting more than 120 min after time of injury - Patients who have received more than 2000 ml of intravenous fluids prior to emergency department arrival - Patients with burns > 5% of their body surface area - Patients taking anticoagulant medication other than aspirin (< 650 mg/day) - Patients with a known bleeding diathesis - Patients with moderate to severe liver disease (Child-Pugh B or C3) |

**Supplemental Table 2. Measurements and missing data**

| **Outcome measure** | **Measurement available, n (%)** | **Reason not available** |
| --- | --- | --- |
| ADAMTS13 conformation index | 39 (100) | n/a |
| ADAMTS13 antigen | 39 (100) | n/a |
| ADAMTS13 activity | 34 (87) | Insufficient sample volume: 5 |
| ROTEM | 22 (56) | Assay not performed: 7*  Technical error: 10** |
| Mortality | 39 (100) | n/a |
| Lactate | 29 (74) | Assay not performed: 10 |
| Base deficit | 37 (95) | Assay not performed: 2 |
| Hb | 38 (97) | Assay not performed: 1 |
| Platelet count | 33 (85) | Assay not performed: 6 |
| Von Willebrand factor | 18 (46) | Insufficient sample volume: 21 |

**May be due to lack of blood sample, failure to save data (in case of ROTEM) or logistical reasons. **Error code in ROTEM data export. May be due to insufficient sample volume, error with assay procedure or premature termination of the assay.*

**Supplemental Table 3. ADAMTS13 auto-antibodies were undetectable in trauma patients with an conformation index >0.5.**

| ADAMTS13 conformation index | ADAMTS13 activity (%) | ADAMTS13 antigen (µg/mL) | anti-ADAMTS13 auto-antibodies (%) |
| --- | --- | --- | --- |
| 0.63 | **298.98** | 0.3 | <6.7 |
| 0.80 | **443.87** | 0.43 | <6.7 |
| 1.55 | **641.06** | 0.37 | <6.7 |
| 2.18 | **902.26** | 0.31 | <6.7 |

**Supplemental Table 4. Rotational thromboelastometry parameters within the trauma cohort**

|  | **No shock** | **Shock** | | |
| --- | --- | --- | --- | --- |
|  | **Closed ADAMTS13** | **Closed ADAMTS13** | **Truncated ADAMTS13** | **p value*** |
| EXTEM, clotting time (s) | 51 (47 – 66) | 65 (58 – 75) | 218 (68 – 308) | <0.01 |
| EXTEM, max clot firmness (mm) | 59 (54 – 71) | 62,5 (57 – 63)) | 12 (5 – 46) | <0.01 |
| EXTEM, max lysis (%) | 15 (12 – 20) | 6 (2 – 10) | 98 (43 – 100) | <0.01 |
| FIBTEM, clotting time (s) | 53 (39 – 66) | 66 (58 – 69) | 144 (53 – 287) | 0.53 |
| FIBTEM, max clot firmness (mm) | 17 (16 – 20) | 12 (11 – 15) | 6 (5 – 15) | 0.06 |
| FIBTEM, max lysis (%) | 12 (6 – 19) | 1 (0– 7) | 85 (62 – 100) | <0.01 |

Trauma patients are stratified based on the presence of shock (BD > 5 or activation of the massive transfusion protocol [MTP]). Patients in shock were further stratified based on ADAMTS13 conformation (closed or truncated). *The p-value is based on a comparison between the closed ADAMTS13 group and the truncated ADAMTS13 group (Mann–Whitney U).

**Supplemental Table 5. Cause of mortality.**

|  | Closed ADAMTS13 | Truncated ADAMTS13 |
| --- | --- | --- |
| Cause of death | **Mortality within 24 h** | |
| Traumatic brain injury, n (%) | **1 (100)** | **2 (50)** |
| Drowning, n (%) |  | **1 (25)** |
| Massive blood loss, n (%) |  | **1( 25)** |
| Cause of death, n (%) | **Mortality 24h - 28 d** | |
| Traumatic brain injury, n (%) | **3 (60)** |  |
| Pericardial tamponade, n (%) |  | **1 (100)** |
| Organ failure, n (%) | **2 (40)** |  |

***
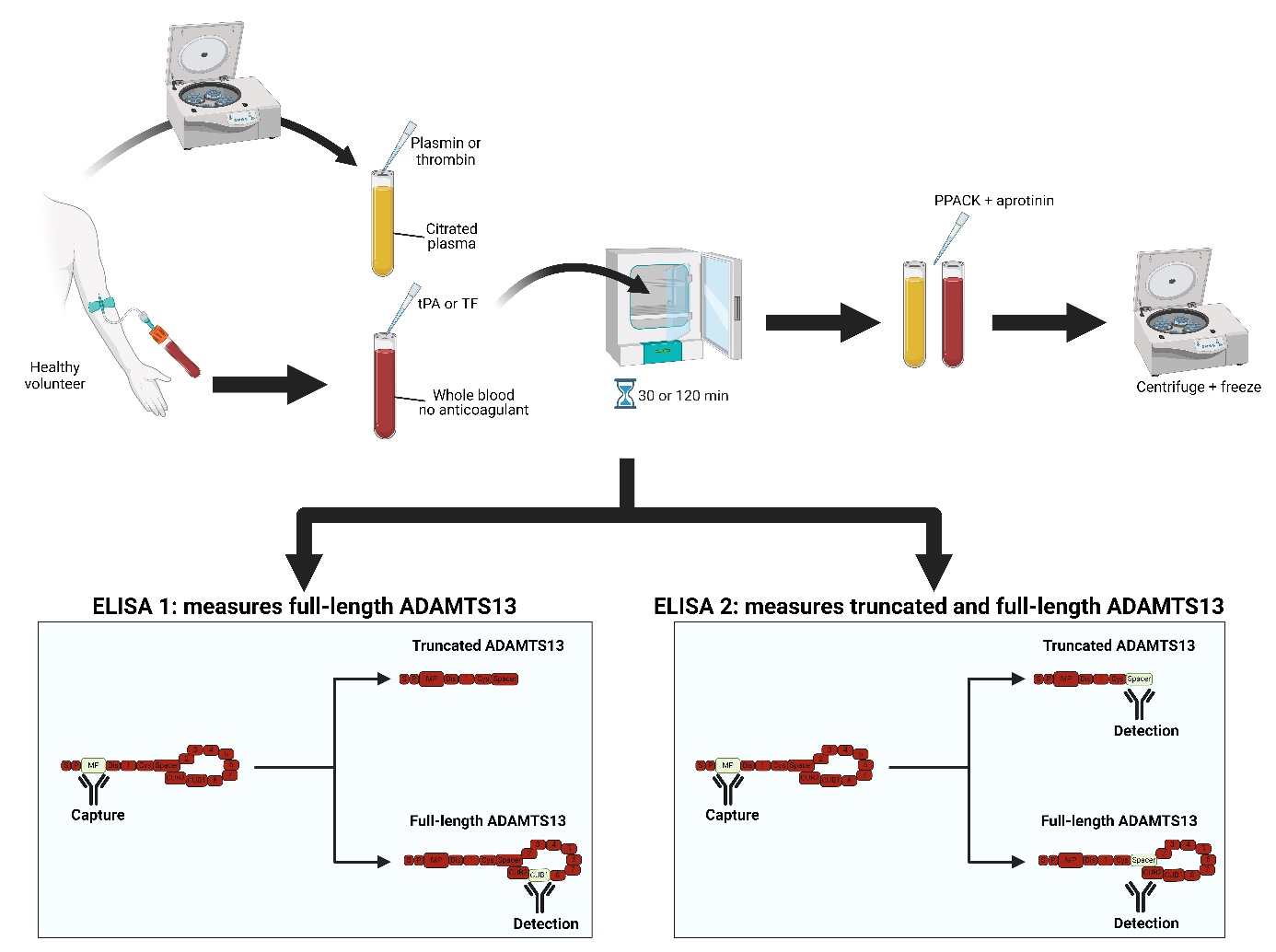
Supplemental Figure 1. Overview of the in vitro incubation experiment.*** *Blood was collected from six healthy volunteers in glass tubes without anticoagulant or tubes with citrate. The blood without anticoagulant was incubated immediately with tissue plasminogen activator (tPA) or tissue factor (TF). The citrated blood was centrifuged twice at 2500G and subsequently incubated with plasmin or thrombin. Incubation was done for 30 or 120 min at 37* *°C. After incubation PPACK and aprotinin were added and all tubes were centrifuged twice at 2500 G to obtain serum (whole blood incubation conditions) or to get rid of any fibrin polymers that formed (in the thrombin condition). Samples were then snapfrozen in liquid nitrogen and stored at -80 °C until being used for enzyme linked immunosorbent assay (ELISA) to measure ADAMTS13 antigen. Two distinct ELISAs were performed (i.e. with different detection antibodies), to distinguish between full length and truncated ADAMTS13 variants.*

***Supplemental Figure 2. Fibrin formation assay parameters****. CLT-50% = 50% clot lysis time; the time in minutes from the midpoint from baseline to maximum turbidity, to the midpoint in the transition from maximum turbidity to the final turbidity. OD-20min = optical density at 20 minutes from the start of measurement. AUC = area under the curve; integral calculated from lag time to the end of first peak (i.e. until 100% lysis time).*

***
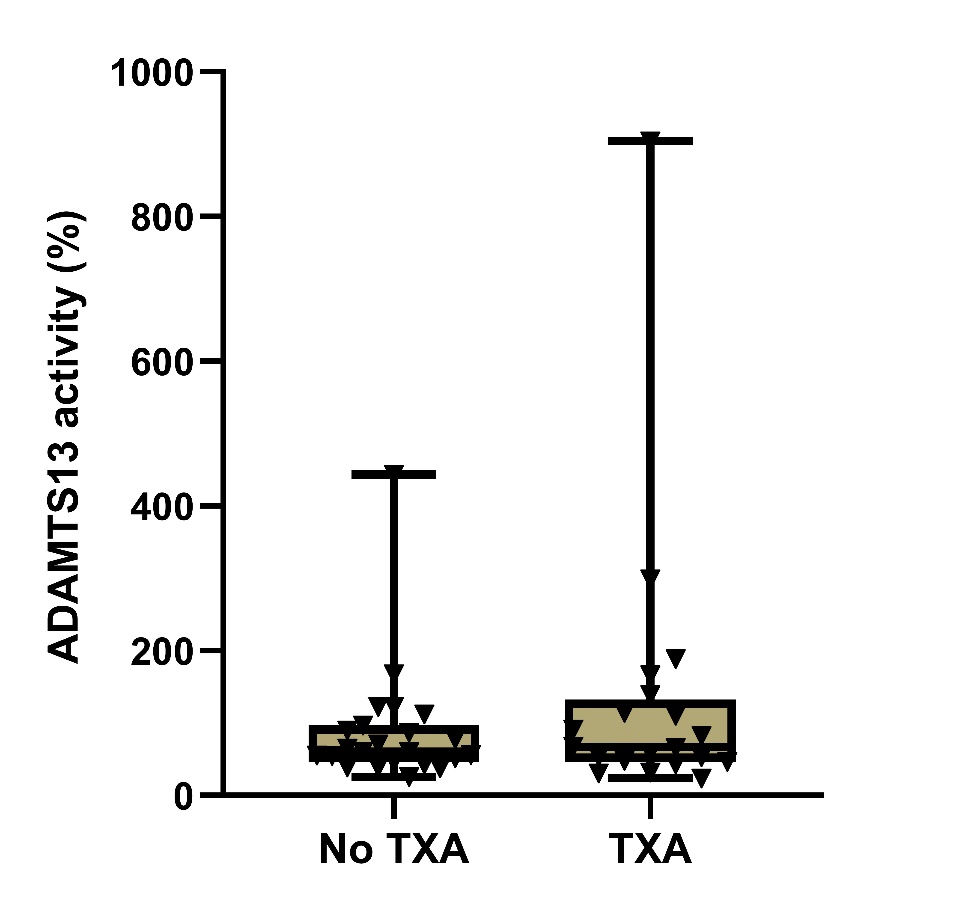
Supplemental Figure 3. ADAMTS13 activity did not differ in patients that received tranexamic acid (TXA) and patients that did not.*** *Data presented as boxplot with all datapoints.*


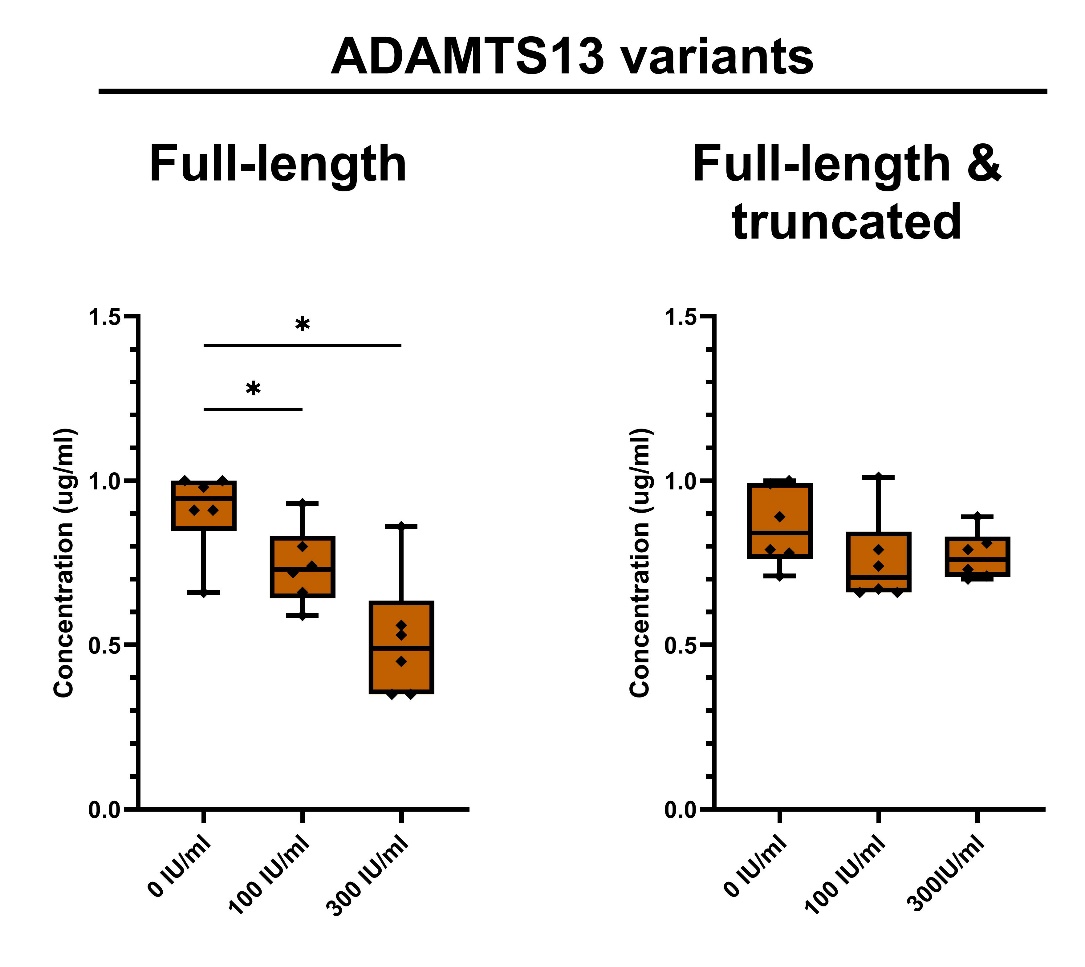


**Supplemental figure 4. ADAMTS13 is truncated in the presence of uPA.** Incubation of plasma with urokinase-type plasminogen activator for 120 minutes resulted in a dose-dependent decrease in full length ADAMTS13 antigen, producing truncated ADAMTS13 with increased activity. Data are presented as boxplot. All data points are shown. * p < 0.05.

**
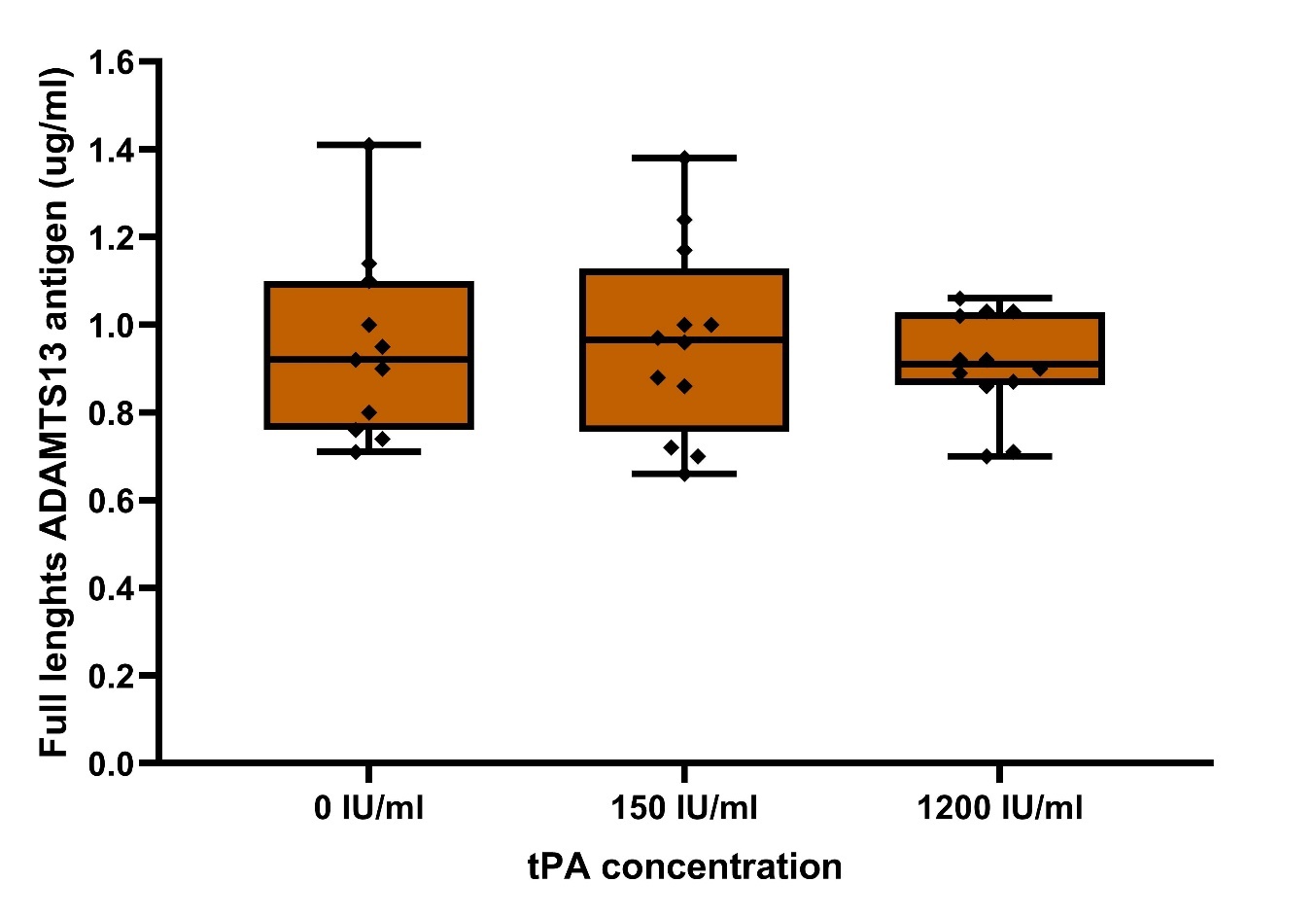
**

**Supplemental figure 5. tPA does not directly influence full-length ADAMST13 antigen.** Incubation of citrated plasma with tissue plasminogen activator (tPA) for 120 minutes did not result in truncation of ADAMTS13. Data are presented as boxplot. All data points are shown.


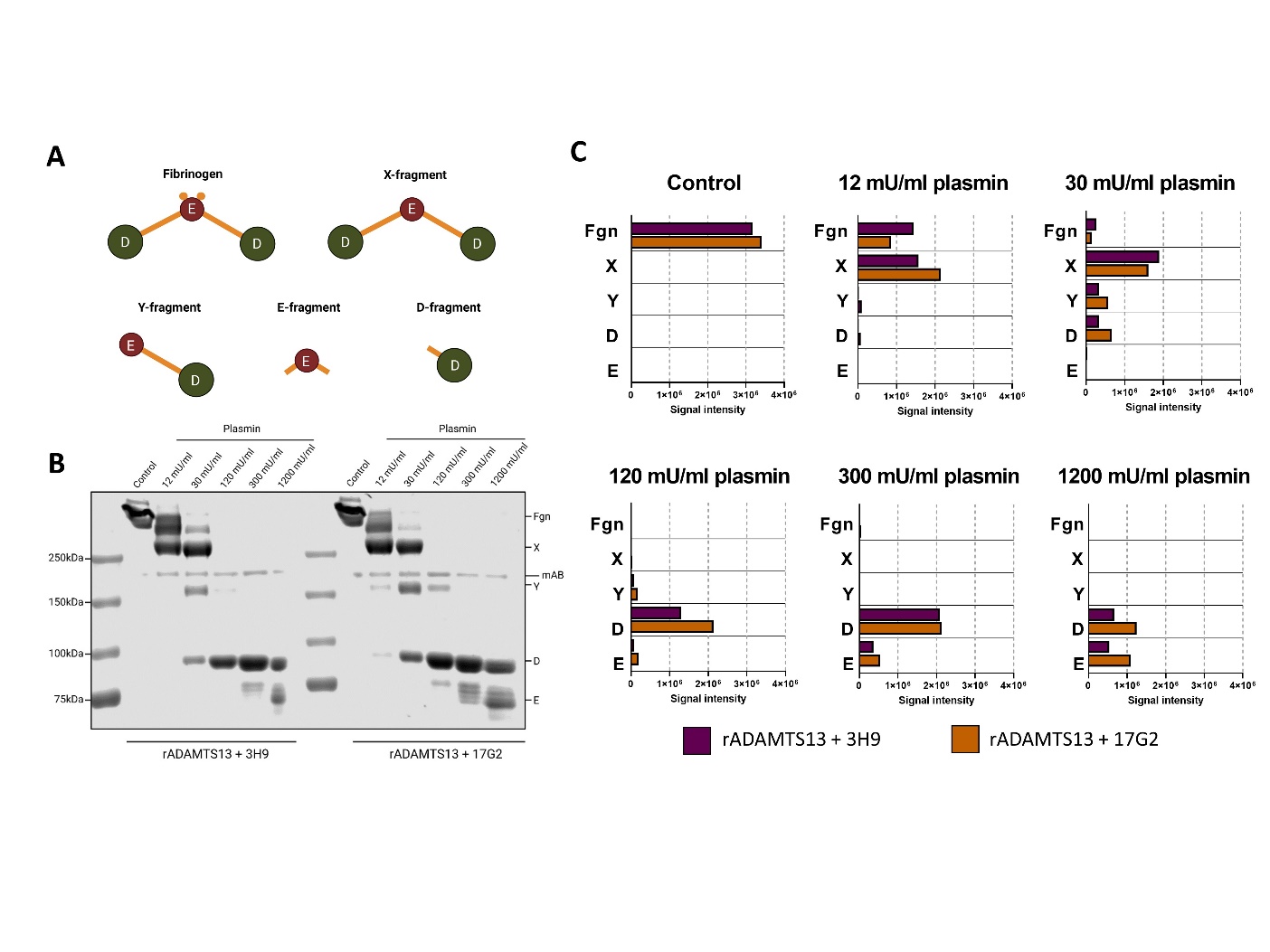


**Supplemental Figure 6. The effect of ADAMTS13 on fibrinogen degradation in isolated conditions A)** Schematic overview of the fibrinogen degradation products. **B,C)** Coomassie blue staining with signal intensity measurement. Incubation of purified fibrinogen with activated recombinant ADAMTS13 (rADAMTS13 + 17G2) results in an increase in fibrin degradation products in the presence of increasing concentrations of plasmin, compared inhibited ADAMTS13 (rADAMTS13 + 3H9).
